# Can Measurement of Ultrasonic Echo Intensity Predict Physical Frailty in Older Adults?

**DOI:** 10.3390/diagnostics13040675

**Published:** 2023-02-11

**Authors:** Yoshihiro Tagami, Keiko Fujimoto, Takaharu Goto, Hideki Suito, Kan Nagao, Tetsuo Ichikawa

**Affiliations:** 1 Department of Prosthodontics and Oral Rehabilitation, Graduate School of Biomedical Sciences, Tokushima University, Tokushima 770-8504, Japan; 2 Department of Oral and Maxillofacial Radiology, Graduate School of Biomedical Sciences, Tokushima University, Tokushima 770-8504, Japan

**Keywords:** echo intensity, tongue pressure, frailty, grip strength, Kihon Checklist, older adults

## Abstract

Echo intensity (EI) of the tongue in ultrasonography is an easy and quantitative assessment of tongue function. Elucidating the relationship between EI and frailty, is expected to aid in the early detection of frailty and oral hypofunction in older adults. We assessed tongue function and frailty in older outpatients visiting a hospital. The subjects were 101 individuals aged 65 or older (35 men and 66 women, mean age 76.4 ± 7.0 years). Tongue pressure and EI were measured as assessments of tongue function and grip strength, and Kihon Checklist (KCL) scores were measured as assessments of frailty. In women, a significant correlation was not found between the mean EI and grip strength, whereas a significant correlation was noted between each score of the KCL and the mean EI; the scores increased as the mean EI increased. A significant positive correlation was found between tongue pressure and grip strength, but no significant correlation was observed between tongue pressure and the KCL scores. In men, no significant correlation was found between tongue assessments and frailty, except for a significant positive correlation between tongue pressure and grip strength. The results of this study suggest that EI of the tongue is positively associated with physical frailty in women and may be useful for early detection of physical frailty status.

## 1. Introduction

In recent years, the increase in the number of older adults requiring long-term care has become a major social problem in Europe, the United States, and East Asia, particularly Japan. Many of the conditions requiring long-term care are considered to be preceded by a state of “frailty [1]”. Frailty is defined as a state in which motor and cognitive functions deteriorate with age. The coexistence of multiple chronic diseases impairs daily living functions, resulting in the emergence of physical, mental, and social frailty [2,3]. However, daily living functions can be improved and raised with appropriate intervention and support, meaning a state between a healthy state and a state of care that requires support in daily living. Early detection of frailty as a reversible condition and countermeasure against frailty before it becomes an irreversible condition requiring long-term care would lead to a decrease in the time required for long-term care and an extension of healthy life expectancy [4].

A decline in oral function (oral hypofunction) is supposed to occur before frailty [5,6]. This refers to a lower chewing ability, greater tooth loss, an inability to move the tongue skillfully, lower tongue strength, difficulty eating hard foods, and choking easily. Tongue function plays a particularly important role in oral function, and the diagnostic criteria for oral hypofunction include decreased tongue pressure and tongue motor function. These assessments are based on the self-effort behaviors of the patient; therefore, measuring them in patients with dementia or higher brain dysfunction is difficult. Tongue pressure measurement is also difficult in edentulous jaw patients and complete denture wearers because the probe for measuring tongue pressure is grasped by the anterior teeth.

In this study, we focused on the echo intensity (EI) of the tongue upon ultrasonography for an easy and quantitative assessment of tongue function. EI has been reported to indicate changes in muscle quality, as higher levels of EI are associated with an increase in fatty and fibrous tissues in the muscle. Recently, EI has been used as an objective biomarker of age-related changes and disease progression, such as increases in fatty infiltration and fibrosis [7,8]. We also found a relationship between EI and tongue pressure, i.e., the EI of the tongue tends to be higher in patients with lower tongue pressure [9].

Although many reports have shown a relationship between frailty and oral hypofunction, including low tongue pressure [10,11,12], few reports have directly demonstrated a relationship between frailty and the EI of the tongue. Elucidating the relationship between EI and frailty, which is easy to measure and assess, is expected to aid in the early detection of frailty and oral hypofunction. Therefore, in this study, based on the hypothesis that the assessment of tongue function, particularly the echo intensity of the tongue, predicts physical frailty, we aimed to assess tongue function and frailty in older outpatients visiting a hospital.

## 2. Materials and Methods

### 2.1. Subjects

The subjects who visited the Dental Division of Tokushima University Hospital for maintenance between January 2021 and July 2021 and could participate in this study were consecutively included appropriately. Exclusion criteria included individuals who could not follow the instructions of the examiner, who were not competent in the measurements because of serious systemic diseases, and who had maxillofacial defect prostheses. The subjects totaled 101 individuals aged 65 or older (35 men and 66 women, mean age 76.4 ± 7.0 years). Age, sex, and body mass index were recorded using a height and weight meter (Tsutsumi, Tokyo, Japan) as basic characteristics.

This study was approved by the Clinical Research Institutional Review Board of the Ethics Committee of Tokushima University Hospital (Approval No. 3880) and was conducted in compliance with the Declaration of Helsinki. Measurements were carried out after the patients were provided with a sufficient explanation about the study, and their consent was obtained with a signature.

### 2.2. Tongue Assessment

#### 2.2.1. Echo Intensity of the Tongue

**Figure 1:**
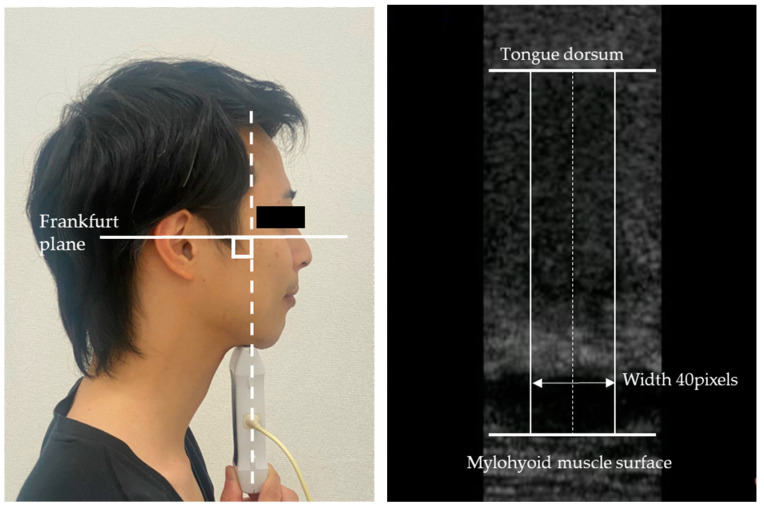
Echo intensity measurement of tongue using ultrasonography. Probe and head position (**left**), and measurement of echo image (**right**).

The EI of the tongue was measured using ultrasonography (Vscan with Dual Probe; GE Healthcare Japan, Tokyo, Japan) according to our previous study [9]. The subjects were seated in a dental chair with the headrest adjusted so that the Frankfurt plane was horizontal to the floor, and the head and back were secured to the backrest to prevent body movement. The ultrasound probe (frequency, 4.0–8.0 MHz; contact face size, 9 × 25 mm; mechanical index, 0.4; thermal index, 0.1) was positioned perpendicular to the Frankfurt plane and in the middle of the bilateral second premolars (Figure 1). The patients were instructed to swallow saliva and take the mandibular rest position. Still images were recorded when the tongue was in a stable position. The distance from the mylohyoid muscle surface to the dorsal surface of the tongue was measured in still images, and the mean EI was measured in 40 pixels wide area across the axis of distance measurement (Figure 1). Image analysis software (ImageJ; NIH, Bethesda, MD, USA) was used to analyze the EI on the ultrasound images, and tongue quality was evaluated in the range of 8 bits (0 [black] to 255 [white]). One trained examiner measured ultrasound images three times, measuring the mean EI for each image without prior information. The mean of three measurements was used as a representative value.

#### 2.2.2. Tongue Pressure

**Figure 2:**
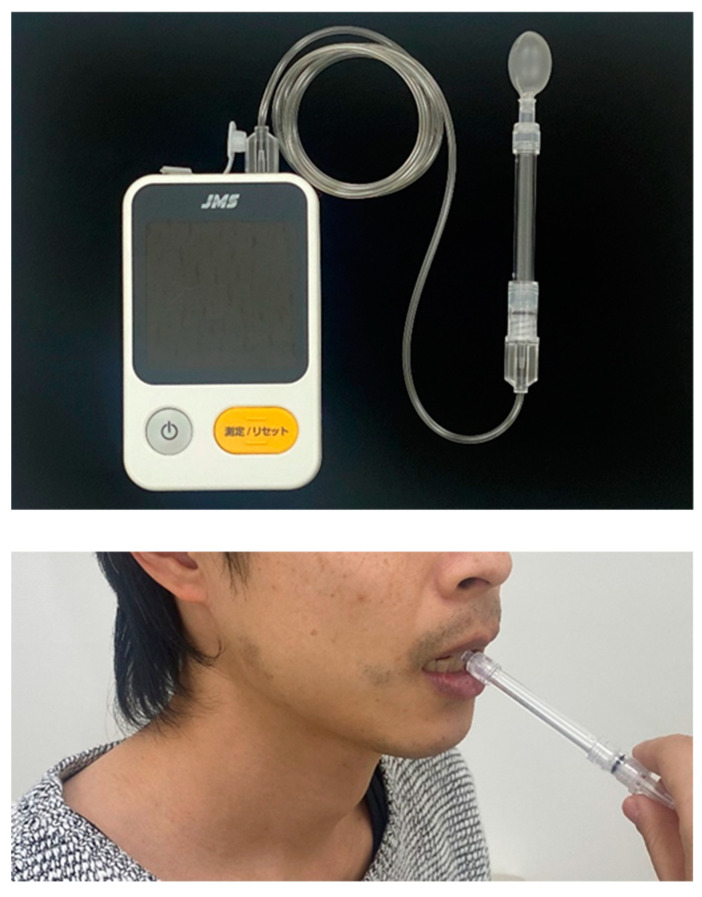
JMS tongue pressure measurement device (**top**), and measurements of tongue pressure (**bottom**).

Tongue pressure was measured using a JMS tongue pressure measurement device (JMS, Hiroshima, Japan). The measurement was performed after calibration outside the oral cavity and by placing a tongue pressure probe between the tongue and palate. The rigid ring of the tongue pressure probe was lightly held with the incisors, and the participants were instructed to raise their tongue with maximum force against the palate for 7 s (Figure 2). The displayed maximum pressure measured using the digital tongue pressure measurement device was recorded. Each measurement was performed thrice, with a break, and the mean value was used as the representative value.

### 2.3. Frailty Assessment

#### 2.3.1. Kihon Checklist

The Kihon Checklist (KCL), commonly used in medical and nursing practice in Japan [13], was used as a subjective assessment of frailty. KCL consists of 25 questions (Table 1), with responses restricted to either “yes” or “no”. In this study, the total score (number of applicable questions, #1–25), lifestyle score (number of applicable questions, #1–20), and physical function score (number of applicable questions, #6–10) were evaluated.

#### 2.3.2. Grip Strength

Grip strength was measured using a grip strength meter (MP-HBM03-BK; Tokyo, Japan) as an objective frailty assessment. It was measured once on each side, and the mean value was recorded as grip strength.

### 2.4. Statistical Analysis

The Mann–Whitney U test was performed to analyze differences in age, tongue pressure, mean EI, KCL, and grip strength between men and women. Spearman’s correlation analysis was used to analyze the relationship between tongue assessments (tongue pressure and EI) and frailty (KCL score and grip strength). SPSS^®®^ (version 25.0; SPSS Inc., Chicago, IL, USA) was used for all statistical analyses, and the risk rate was set at a significance level of less than 5%.

## 3. Results

Table 2 shows the summary of measurements. The subjects were 35 men (mean age 76.3 ± 7.4 years) and 66 women (mean age 76.4 ± 6.8 years). Significant differences were not found in age between men and women.

The mean EI of the tongue was 33.7 ± 6.6 in men. It was 38.9 ± 6.1 in women, which was significantly higher. Tongue pressure was 29.4 ± 9.5 kPa in men and 31.0 ± 8.0 kPa in women. No significant differences were found in tongue pressure between men and women. Many subjects showed no decline in tongue muscle strength.

Regarding frailty assessment, grip strength was 32.5 ± 6.1 kgf in men and 19.7 ± 4.8 kgf in women. Grip strength was significantly higher in men than in women. Although grip strength in both men and women was lower than the Japanese average by generation, many subjects showed higher grip strength than the criterion for frailty, which is less than 28 kgf for men and 18 kgf for women. The subject group was speculated to include many who had muscle strength just starting to decline.

In the KCL, the total score was 5.8 ± 3.7 in men and 6.1 ± 4.0 in women. The lifestyle score was 4.8 ± 2.9 in men and 4.9 ± 3.1 in women, and the physical function score was 1.1 ± 1.0 in men and 1.7 ± 1.5 in women. The physical function score in women was higher than that in men, but no significant difference was found in the other scores between men and women.

Table 3 shows the relationship between tongue function and frailty according to sex. In women, a significant correlation was not found between the mean EI and grip strength, whereas a significant correlation was noted between each score of the KCL and the mean EI (total score, ρ = 0.383; lifestyle score, ρ = 0.398; physical score, ρ = 0.293); the scores increased as the mean EI increased. A significant positive correlation was found between tongue pressure and grip strength (ρ = 0.250), but no significant correlation was observed between tongue pressure and each score of the KCL. In men, no significant correlation was found between tongue assessments and frailty, except for a significant positive correlation between tongue pressure and grip strength (ρ = 0.404).

## 4. Discussion

In Japan, the social environment is dramatically changing with the rise of the super-aged society. Medical and nursing care costs are increasing due to extended life span, and the lack of care workers is a serious problem. Older adults need to remain physically and mentally healthy to live independently. Early detection of frailty, the prodromal state of the need for nursing care, and the extension of healthy life expectancy are important.

A decline in oral function is generally predicted to begin before the stage of frailty [12]. Tongue function, which is related to mastication, swallowing, and articulation, is particularly important in oral function and closely related to the function of daily living in older people [14,15,16]. Tongue pressure and oral diadochokinesis assess tongue function in older adults [5]. Previous studies reported a significant correlation between tongue pressure and grip strength, which is one of the criteria for frailty [17,18,19], and the measurement of tongue pressure is suggested to be effective for assessing frailty. EI measurement from ultrasound images of the tongue was effective, as a significant correlation was found between the EI of the tongue and tongue pressure, and it was less stressful than the tongue pressure measurement [9].

Grip strength as an objective assessment and KCL as a subjective assessment were used to assess frailty. Grip strength is used in both Fried’s definition of frailty [2] and the definition of sarcopenia [20] and is a simple and easy objective test. The KCL is a commonly used screening questionnaire for the comprehensive assessment of frailty in Japan, and its reliability and validity have been documented [21,22]. The 25 questions in the KCL are classified into seven domains: lifestyle of daily living, physical function, nutrition, oral function, socialization, cognitive function, and depressed mood. Some items are significantly affected by environmental and psychological factors. Therefore, the total score and the scores of two domains (lifestyle and physical function) were used to assess physical frailty in this study.

Most of the subjects who participated in this study were relatively healthy older adults who could visit hospitals by themselves and undergo the assessments of body mass index, tongue pressure, grip strength, and KCL scores. Because of their relatively good health and independent function, the participants were considered an appropriate patient group to evaluate whether tongue function assessments can predict frailty.

In the male group, no relationship was observed between tongue assessment and frailty. In the female group, EI was significantly associated with the three KCL scores, and tongue pressure was significantly associated with grip strength. Regarding the relationship between grip strength as an objective assessment of frailty and KCL scores as a subjective assessment of frailty, no significant relationship was found in men, whereas significant in women for all three measures, especially for motor function scores. According to the Ministry of Health, Labour, and Welfare’s National Survey of Basic Living Conditions in Japan, women requiring nursing care is likely due to frailty, joint failure, and fall-related fractures, suggesting that muscle and bone-related decline is more likely to affect women [23].

Skeletal muscle studies have reported that qualitative decline precedes muscle mass loss in patients with hip osteoarthritis [24] and that age-related changes in muscle quality occur at an earlier age than the loss of muscle quantity in community-dwelling older people [25]. Qualitative decline precedes muscle mass loss and functional decline in this group. Therefore, it is expected that qualitative assessment of changes in muscle function predicts changes in muscle. It is reported that tongue thickness was linearly associated with tongue pressure [26]. Our previous study also showed that the EI decreased with increased tongue thickness [9]. An EI was associated with a subjective symptom of frailty, and tongue pressure was associated with an objective sign of grip strength. It is suggested that the EI would increase in the early stage of the frailty process while tongue pressure would decrease.

The present study is a cross-sectional observational study. The sample subjects were motivated, comparatively healthy older individuals. Study limitations include a sampling size difference between men and women, which could produce some sampling bias. In addition, only the grip strength was used as a diagnostic criterion of physical frailty. These limitations must be considered when interpreting this result. However, our findings show that an increase in EI, which would indicate a decline in tongue function, is a greater predictor than decreased tongue pressure for the onset of physical frailty, indicating a high likelihood that EI of the tongue predicts physical frailty. Further, 106 subjects were required as the sample size by a prespecified test; 101 subjects consented to the study within the study period. The power of the post-hoc test was 0.86, which was appropriate for the sample size. Since this study was a cross-sectional study without a multivariate analysis, a longitudinal study is needed to determine that the EI of the tongue is a predictor of frailty.

## 5. Conclusions

The results of this study suggest that EI of the tongue is positively associated with physical frailty in women and may be useful for early detection of physical frailty status.

## Figures and Tables

**Table 1:** Kihon Checklist (**top**) and its seven domains (**bottom**).

No.	Question			Answer	
1	Do you go out by bus or train by yourself?			0. Yes	1. No
2	Do you go shopping to buy your daily necessities by yourself?			0. Yes	1. No
3	Do you manage your own deposits and savings at the bank?			0. Yes	1. No
4	Do you sometimes visit your friends?			0. Yes	1. No
5	Do you tum to your family or friends for advice?			0. Yes	1. No
6	Do you normally climb stairs without using handrails or walls for support?			0. Yes	1. No
7	Do you normally stand up from a chair without any aids?			0. Yes	1. No
8	Do you normally walk continuously for 15 min?			0. Yes	1. No
9	Have you experienced a fall in the past year?			1. Yes	0. No
10	Do you have a fear of falling while walking?			1. Yes	0. No
11	Have you lost 2 kg or more in the past 6 months?			1. Yes	0. No
12	Height: cm, weight: kg, BMI: kg/m^2^ If BMI is less than 18.5, this item is scored			1. Yes	0. No
13	Do you have any difficulties eating tough foods compared to 6 months ago?			1. Yes	0. No
14	Have you choked on your tea or soup recently?			1. Yes	0. No
15	Do you often experience having a dry mouth?			1. Yes	0. No
16	Do you go out at least once a week?			0. Yes	1. No
17	Do you go out less frequently compared to last year?			1. Yes	0. No
18	Do your family or your friends point out your memory loss?E.g., “You always ask the same question over and over again.”			1. Yes	0. No
19	Do you make a call by looking up phone numbers?			0. Yes	1. No
20	Do you find yourself not knowing today’s date?			1. Yes	0. No
21	In the last two weeks have you felt a lack of fulfilment in your daily life?			1. Yes	0. No
22	In the last two weeks have you felt a lack of joy when doing the things you used to enjoy?			1. Yes	0. No
23	In the last two weeks have you felt any difficulty in doing what you could do easily before?			1. Yes	0. No
24	In the last two weeks have you felt helpless?			1. Yes	0. No
25	In the last two weeks have you felt tired without a reason?			1. Yes	0.No
	**Domains**	**Relevant Questions**	**Cut-Off Point with/without Each Risk**		
	Lifestyle	#1–20	Ten or more negative answers		
	Physical function	#6–10	Three or more negative answers		
	Nutrition	#11, #12	Negative answers to both questions		
	Oral function	#13, #14, #15	Two or more negative answers		
	Socialization	#16, #17	An answer of “No” to #16		
	Cognitive function	#18, #19, #20	One or more negative answers		
	Depressive mood	#21–25	Two or more negative answers		

**Table 2:** Assessment results of tongue function and frailty (means ± standard deviations).

Variable	Men (*n* = 35)	Women (*n* = 66)	*p*-Value
Age (years)	76.3 ± 7.4	76.4 ± 6.8	0.957
BMI	23.4 ± 2.5	22.9 ± 3.3	0.544
Grip strength (kgf)	32.5 ± 6.1	19.7 ± 4.8	*p* < 0.01 *
Tongue pressure (kPa)	29.4 ± 9.5	31.0 ± 8.0	0.420
Echo intensity	33.7 ± 6.5	38.9 ± 6.2	*p* < 0.01 *
**Kihon Checklist**			
Total score	5.8 ± 3.7	6.1 ± 4.0	0.909
Lifestyle score	4.8 ± 2.9	4.9 ± 3.1	0.906
Physical function score	1.1 ± 1.0	1.7 ± 1.5	0.060

* *p*-value < 0.05.

**Table 3:** Spearman’s correlation coefficients (Spearman’s rho) between assessments of tongue function and frailty.

	Frail Condition	Grip Strength	Kihon Checklist		
Tongue Function			Total Score	Lifestyle Score	Physical Function Score
Women					
Tongue pressure		0.250 *	−0.134	−0.171	−0.154
Echo intensity		−0.060	0.383 **	0.398 **	0.293 *
Men					
Tongue pressure		0.404 *	−0.219	−0.259	−0.042
Echo intensity		−0.169	0.092	0.060	−0.097

* *p*-value < 0.05, ** *p*-value < 0.01.

## Data Availability

The data presented in this study are available on request from the corresponding author.
